# Ultrasound and nanomaterial: an efficient pair to fight cancer

**DOI:** 10.1186/s12951-022-01243-w

**Published:** 2022-03-18

**Authors:** Edouard Alphandéry

**Affiliations:** 1 Sorbonne Université, Muséum National d’Histoire Naturelle, UMR CNRS, 7590, IRD, Institut de Minéralogie, de Physique des Matériaux et de. Cosmochimie, IMPMC, 75005 Paris, France; 2 Nanobacterie SARL, 36 boulevard Flandrin, 75116 Paris, France; 3 Institute of Anatomy, UZH University of Zurich, Instiute of Anatomy, Winterthurerstrasse 190, 8057 Zurich, Switzerland

**Keywords:** Nanomaterials, Nanotechnology, Nanomedicine, Nano-oncology, Cancer, Ultrasound, High intensity ultrasounds, Contrast agent, Sonodynamic therapy

## Abstract

Ultrasounds are often used in cancer treatment protocols, e.g. to collect tumor tissues in the right location using ultrasound-guided biopsy, to image the region of the tumor using more affordable and easier to use apparatus than MRI and CT, or to ablate tumor tissues using HIFU. The efficacy of these methods can be further improved by combining them with various nano-systems, thus enabling: (i) a better resolution of ultrasound imaging, allowing for example the visualization of angiogenic blood vessels, (ii) the specific tumor targeting of anti-tumor chemotherapeutic drugs or gases attached to or encapsulated in nano-systems and released in a controlled manner in the tumor under ultrasound application, (iii) tumor treatment at tumor site using more moderate heating temperatures than with HIFU. Furthermore, some nano-systems display adjustable sizes, i.e. nanobubbles can grow into micro-bubbles. Such dual size is advantageous since it enables gathering within the same unit the targeting properties of nano bubbles via EPR effect and the enhanced ultrasound contrasting properties of micro bubbles. Interestingly, the way in which nano-systems act against a tumor could in principle also be adjusted by accurately selecting the nano-system among a large choice and by tuning the values of the ultrasound parameters, which can lead, due to their mechanical nature, to specific effects such as cavitation that are usually not observed with purely electromagnetic waves and can potentially help destroying the tumor. This review highlights the clinical potential of these combined treatments that can improve the benefit/risk ratio of current cancer treatments.

**Graphical Abstract:**

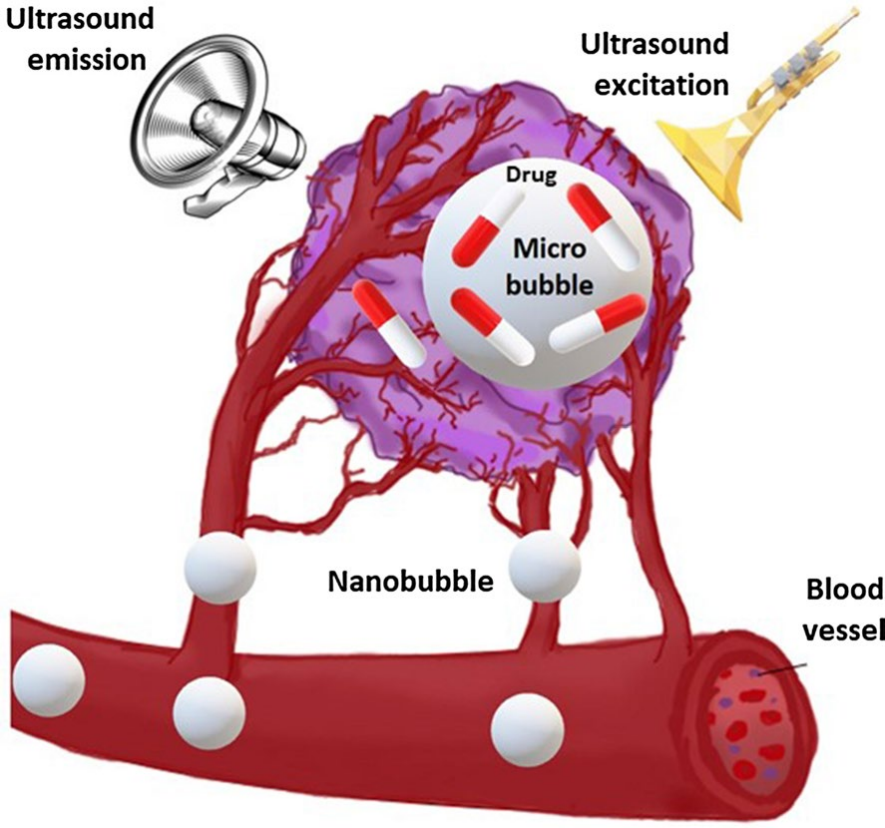

## Introduction

Cancer mortality rate increases with population aging or exposure to risk factors such as alcohol, tobacco consumption, obesity, or the presence of a pandemic like COVID-19, which limits access to patient care, [1]. These trends tend to be particularly pronounced in the least medicalized countries and for advanced or difficult to treat cancers, [2]. While nanotechnology often appears as an all-encompassing and abstract term, it may in fact have the potential to significantly lower this rate, e.g. by making cancer treatments less expensive and thus accessible for people living in countries without social security, by making certain heavy operations such as general anesthesia unnecessary, or by improving the benefit to risk ratio of cancer treatments, [3]. Among the different types of nanotechnologies that could be considered for such purpose, the combination of nano-systems with ultrasounds is especially appealing. Indeed, it gathers a series of advantages. First, it might allow localized cancer treatment at tumor site by enabling therapeutic nano-systems to specifically target tumors via passive, magnetic, or active targeting, [4]. Second, it can also improve the sensitivity of tumor detection by enabling either high resolution ultrasound imaging, e.g. nano/micro bubbles can help visualizing blood vessels irrigating the tumors, [5], or the combination of standard US detection methods with other imaging methods such as PA, MRI, or CT, [6]. Ultrasounds can in some cases help to achieve such targeting by permeabilizing certain barriers such as those of the brain or skin through which nano-systems should diffuse before reaching the tumor, [7, 8]. They can also serve as a source of excitation to activate nano-systems in the tumor in several manner, e.g. through cellular internalization of nano-systems in tumor cells, [9], or via thermal or immune activation, [10], or controlled drug release, [11]. This review highlights the advantages of these combined cancer treatments compared with more commonly used un-paired therapeutic or diagnostic ultrasounds.

### Ultrasound parameters used for cancer therapy

Ultrasounds are mechanical waves that oscillate periodically at a frequency f, which is larger than that of audible sounds, i.e. f > 20 kHz. These waves are usually produced by a transducer, which converts an electric signal in a mechanical displacement. Compared with other types of radiations such as lasers, ultrasounds present the advantage of penetrating more deeply in tissues, leading to their non-invasive use in humans. One can distinguish high intensity focused ultrasounds (HIFU), operating at high intensities i, i.e. i ~ 0.1–10 kW/cm^2^, [12], from low intensity ultrasounds (LIU), which are often less focused than HIFU, and generate ultrasounds of lower intensity, i.e. i < 1–5 W/cm^2^, [13]. On the one hand, HIFU enables to specifically target a tumor region and to rapidly reach the temperature of thermal ablation of typically 80 °C in this region, resulting in coagulative tumor cell death, [14]. While HIFU is relatively well suited to treat small tumors, it faces some difficulties for the treatment of large or hyper-vascularized tumor volumes, for which numerous heating spots may be requested for efficient treatment, necessitating a long treatment time and the use of numerous MRI images to locate the various emplacements of the tumor region that need to be heated, [15], possibly using volumetric heating techniques, [16]. On the other hand, LIU may treat with one application a larger portion of the tumor volume than HIFU but may not enable reaching the temperature of thermal ablation, [17]. For safety reasons, ultrasound imaging should be carried out at intensities kept below ~ 0.05–0.50 W/cm^2^ to avoid heating tissues, [18]. The basis of this recommendation relies on the analysis of specific cases such as fetus imaging, which obviously requires the utmost safety considerations. While designing ultrasound imaging apparatus for tumor tissue, it is not certain that the same rules would apply. With regard to ultrasound frequencies f, they can be divided between relatively high frequencies of typically 3–60 MHz used in medical diagnosis, [19, 20], medium frequency of 0.7–3.0 MHz serving in therapeutic medicine, [21], and low frequency of 20–200 kHz, which can improve drug delivery efficacy at relatively large penetration depth, [22]. To reach a desired outcome, the ultrasound frequency and intensity should be adjusted skillfully, e.g. to increase US penetration depth, US frequency may be reduced, while to enhance US heating, US intensity may be increased, where US frequencies and intensities remain within a range of acceptable values in the medical field. In addition to frequency and intensity, other ultrasound parameters such as ultrasound pressure (MPa), mechanical index, pulse length, pulse repetition frequency, duty cycle, total ultrasound application time, have been adjusted to yield specific ultrasound properties in an organism, [23].

### Different types of nano-systems used for cancer treatment in the presence of ultrasounds

To improve the efficacy of therapeutic or diagnostic ultrasound, nano-systems acting as sonosensitizer or contrast agents were introduced. Concerning sonosensitizers, they are often described as ROS enhancer, a view inspired from the definition of photosensitizers, which produce ROS under laser light irradiation. Since nano-systems exposed to ultrasounds may trigger other mechanisms such as cavitation, heating, or mechanical displacements, I consider here that nano-systems are sonosensitizers when they enhance various types of sono-induced mechanisms, not only ROS production.

**Fig. 1:**
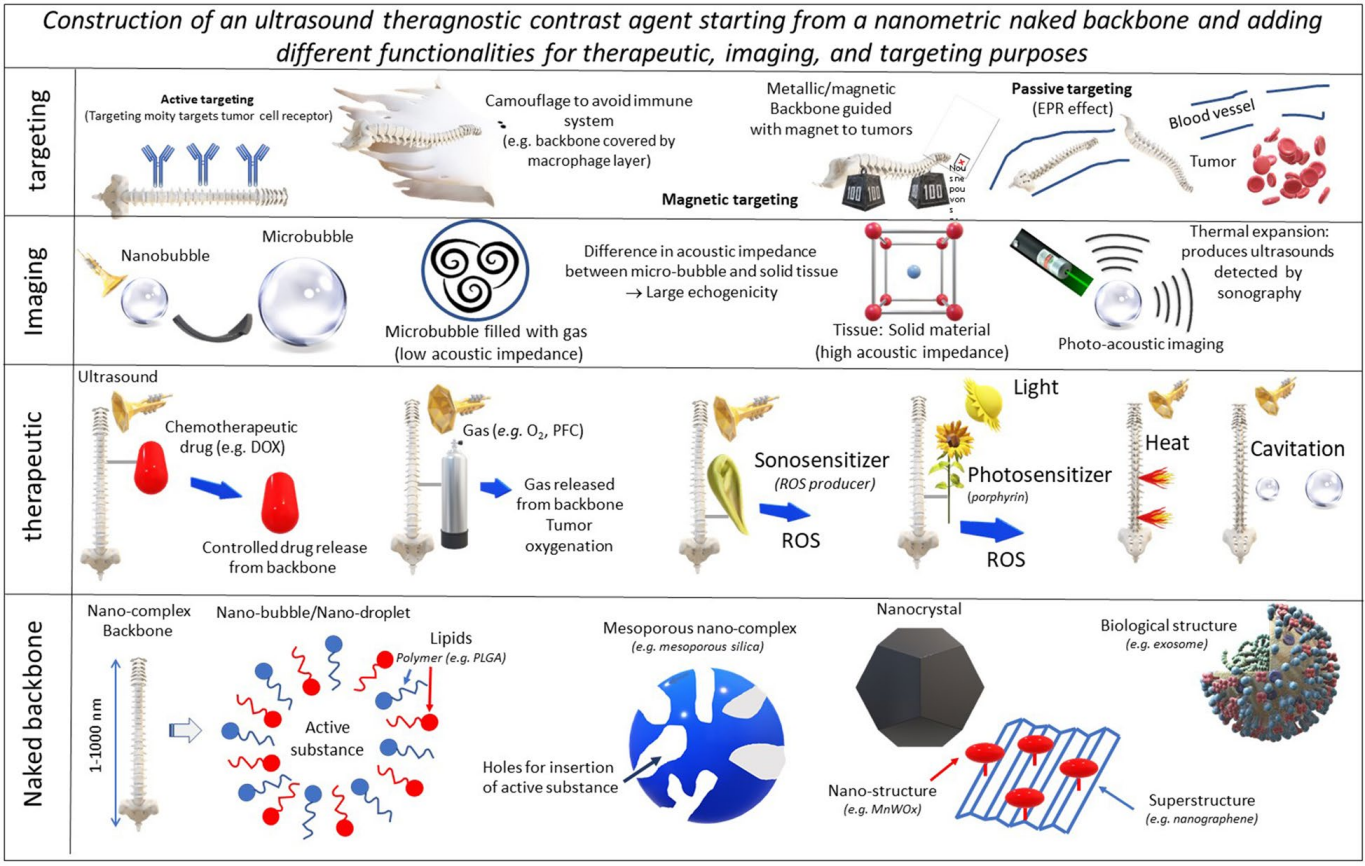
A schematic diagram representing the various types of nano-systems (NS) that have been used for cancer treatment/diagnosis. NS are presented in a hierarchical order, starting from a backbone consisting of nano-bubbles/nano-droplets, mesoporous nano-complexes, nanocrystals, biological nanostructures, to which one or several functional element(s) is/are added. Such functional elements provide the backbone with a therapeutic, imaging, or targeting activity. In some cases, another radiation than ultrasound can be used, such as laser for PDT/PTT treatment or PA imaging, or a magnetic field to yield magnetic targeting when the backbone is made of metallic elements. A unique feature of these nano-systems, which is not often encountered in nano-systems not excited by ultrasounds, lies in their gaseous content, which can either be used for cancer treatment, e.g. through O_2_ release to fight against tumor hypoxia, or for tumor imaging, e.g. by using microbubbles with a different acoustic impedance from that of the tissular environment

Figure 1 shows a schematic representation of the different nano-systems (NS) considered for cancer treatment in the presence of ultrasound applications. It highlights the conception of NS, which are fabricated starting from a nano-metric backbone associated with different functional elements, which improve their therapeutic, imaging, or targeting efficacy. Furthermore, Table 1 summarizes the properties of these materials such as their composition, their average size, their use as diagnostic and/or therapy tools. In general, the backbone consists of the following entities: (i) nano-bubbles, [24–26], or nano-droplets, [27–31, 52], i.e. hollow nanometric spheres filled with gases or liquids, whose external surface is stabilized by polymers such as PMMA, PLGA, [30], or lipids or phospholipids, [32–40, 130], (ii) silica NP with different levels of porosity or meso-porosity, [40–44, 130], (iii) inorganic NP in the form of nanocrystals or porous structures such as gold NP, [45, 46], iron oxide NP, [45, 47–49], TiO_2_ NP, [50], Au-TiO_2_ NP, [51], MnOx NP, [52], bismuth NP, [53], ZnO NP, [54], (iv) natural NP such as those composed of heme-based pigment biliverdin, [55], or albumin, [36–38], (v) certain biological structures such as exosomes, [56], membrane vesicles, [57], protein vesicles of bacterial origin, [58, 59], polymersome, [60–62], self-assembled peptides, [63], which are either isolated from their original biological environment or copied from living material through chemical synthesis. In some interesting cases, nano-systems are associated with superstructures either to ensure their stability or sufficient concentration, e.g. for MnWOx NP attached to graphene sheets, [64], or to combine therapeutic and imaging functionalities together, e.g. for therapeutic magnetic NP attached to the surface of echogenic microbubbles, [47]. In general, functional elements are added to the backbone by being either bound to its external surface or incorporated inside its inner core, at concentrations that are theoretically larger than those, which would be obtained in macroscopic drugs with smaller surface/volume ratio. Such association often aims at preventing these functional elements from being degraded by the organism or lost before they reach the tumor. The first type of functional elements, which yields improved therapeutic efficacy, is made of: (i) chemotherapeutic drugs such as DOX, [36–38, 65, 66], Cis-platin, [34, 35], or Docetaxel, [36–38], whose association with nano-systems should result in an enhanced drug concentration in the tumor and a synergy between chemotherapeutic and ultrasound anti-tumor effects, (ii) two types of gases, those which have a direct toxicity towards the tumor such as carbon monoxide (CO), nitric oxide (NO), hydrogen sulfide (H_2_S), hydrogen (H_2_), sulfur dioxide (SO_2_), carbon dioxide (CO_2_) and oxygen (O_2_) that yields tumor oxygenation and enables chemotherapeutic drug to overcome resistance observed under hypoxic tumor environment, [67], (iii) ROS enhancer such as protoporphyrin, [68–71], TAPP, [72], which are able to generate ROS under ultrasound application. The second category of functional elements, which confers to nano-systems their ultrasound contrasting ability, consists of a nanomaterial with a different acoustic impedance than that of its surrounding tumor tissue, hence enabling the acoustic wave to be reflected at nanomaterial surface and then to travel back to be detected. In most cases, it is made of gases such as sulfur hexafluoride (SF_6_) or perfluorocarbon (PFC) contained in nano bubbles often able to expand into more echogenic microbubbles under certain conditions, [73]. Finally, the backbone can also be associated with an agent that favors its accumulation in the tumor. This agent can be a targeting moiety recognizing a tumor cell receptor such as anti-HER2 antibodies, [32], rabies virus glycoprotein peptides targeting neuroblastoma cells, anti-EGFR antibodies, [48, 49], folic acid, [6], cyclic arginine-glycine-aspartic pentapeptide, [71], antibody targeting epiregulin, [74]. It can also consist in a compound such as macrophage membrane, [75], or PEG, [48, 49], enabling nano-systems to avoid being captured by the immune system. In some cases, the backbone itself has certain functionalities, as it is the case for TiO_2_ NP generating ROS under ultrasound application, for magnetic NP guided towards the tumor with a magnet, or for backbones with composition, geometry or size enabling their passive diffusion towards the tumor via the EPR effect, [76]. To facilitate the pharmaceutical fabrication of these nano-systems in a reproducible manner, it may be easier to use a simple backbone which is devoid of a large number of additional functional elements than more complex structures. Some of the nano-systems presented in the literature display several building blocks. For example, NS made of PLGA NC (block 1) coated with a thin Silica layer (block 2) encapsulating perfluorocarbon (block 3), antitumor Ruthenium complex (block 4), and superparamagnetic Fe_3_O_4_ NP (block 5) consist of 5 distinct blocks, [44]. In a pharmaceutical production process, it would be necessary to demonstrate that each block is fabricated and assembled in sufficient quantity and reproducibly, without forgetting that a high purity level, a long-term stability, an endotoxin-free and sterility of the product should also be achieved. Such hard task may be fulfilled by reducing the number of blocks, [77].

**Table 1:** The values of various parameters, i.e. NM composition, NM average sizes, ultrasound frequency/intensity/focusing/MI, and their associated in vitro and in vivo anti-tumor efficacies as well as mechanisms of action, where tumor diagnosis and therapy are covered

NM composition	NM average size	Therapy (Th) Diagnosis (Di)	Ultrasound parameters Intensity (I); Frequency (f); Mechanical index (MI); focused (fo) vs unfocused (ufo)	In vitro/In vivo/ex vivo efficacy	Mechanism of action	References
MNP + DOX at the surface of MB	MB: 0.4–200 µM MNP (Fe_3_O_4_): 50 nm	Th	f = 0.5–2 MHz Oscillation thresold: 0.4 bar Pressure either above or below 0.5 bar Focused transducer	Delivery of MNP + DOX to mouse tumor tissue	Release of MNP/DOX from MB by ultrasounds above oscillation threshold Diffusion of MNP from MB by several 100 µm	[47]
Magnetic Polylactic co-glycolic acid Nanocapsules (MNC) + 5-fluorouracil	70 nm	Di: PA	f = 3 MHz I between 0,3 and 1 W/cm^2^	NA	Release of + 5-fluorouracil from MNC (increasing release with increasing US intensity)	[205]
Phosphilpid NB Phosphilipd MB Self-assembled amphiphilic polymers (hydrophobic core + hydrophilic exterior): MI	MB: 1–4 µm (Definity, CAV) NB: 100–500 nm MI: 10–100 nm	Di	Frequency larger than 20 MHz	NA (review)	1/ NB: diffuse in tumor by EPR, requires pluoronic acid to be echogenic, can transport drugs; 2/ MB: does not diffuse to tumor via EPR, highly echogenic; 3/ MI: diffuse to tumor via EPR; requires specifc phospholipid concentration to be echogenic, unstable	[33]
Nanodroplet (ND) loaded with Iodine	60 nm	Di	B-mode imaging	iv injection of ND in mice bearing Hepatocellular carcinoma	ND targets liver and hepatocyte Precise tumor volume measurement CT + US CA combined	[27]
Silica NC containing NIR-dyes-modified polymers and ultrasmall gold NP	Silica NC: 100 nm	Di	f = 13–24 MHz	Combination of US and PA features of Silica NC validated ex vivo	Silica NC: US CA Gold NP: PA CA	[41]
MB liposomes conjugated with anti-Her2 antibodies loaded with survivin-targeted siRNA and doxorubicin	NA	Th	f = 5–12 MHz MI = 0,61	MB injected in mice bearing LNCaP prostate tumor xenograft followed by US application: tumor cell apoptosis + suppression of survivin expression	Specifically target PC-3 and LNCaP prostate tumor cells; Intra-cellular delivery observed by CM; Delivery of MB by US application; Survivin expression was suppressed in vivo	[32]
Au NP IONP Nano-graphene oxide (NGO)	Au: 6 nm IONP: 10 nm NGO: 22 nm	Th	f = 1 MHz; i = 2 W/cm^2^;	NP brought into presence of CT26 tumors exposed to US: temperature elevation rates of 12.5%, 20.4%, and 37.5% for IONP, NGO, and AuNP	Temperature increase is more pronounced in the presence than absence of NP	[45]
Liposomes containing Cis-platine	100 nm	Th Di	1 MHz 2 W/cm^2^ B-mode observation of ND	Inhibition of 4T1 human breast cancer cells growth by ND exposed to US	Release of 63% of Curcumin under US application; Acoustic droplet vaporization	[34]
Liposomes containing Cis-platin	NA	Th	20 kHz	Treatment of mice bearing C26 footpad murine tumors with NM + US results in negligible tumor growth one moth following treatment compared with untreated mice	Increase in local Cisplatin concentration in C26 footpad murine tumor following NM administration + US application to 70% in US treated tumors compared with 3% in untreated tumors	[35]
DPP-TPA NP	100 nm	Di	Use of a Nexus128 small animal PA imaging system with 680 nm laser (1 W/cm^2^)	PA of xenografted tumors filled with NP	Increase of PA signal with increasing NP concentration	[156]
NB associated with siRNA (NB-siRNA)	625 nm	Th	f = 1 MHz; i = 0.88 W/cm^2^;	Mice bearing U87 GBM tumors injected iv with NB-SiRNA followed by US application: Tumor growth delay (compared with treatment of NB-SiRNA without US)	NB can accumulate in intercellular spaces (due to their nm size); *“Sonoporation”* produced by UTD: increase cell membrane permeability; NB exposed to US: improve siRNA transfection and silencing of targeted genes;	[24]
Hollow mesoporous organosilica NP functionalized with fluorocarbon chains + IR780 (SS) + O_2_	180 nm	Th	f = 1 MHz I = 1 W/cm^2^	Destruction of PANC-1 cells in vitro; Shrinkage of hypoxic PANC-1 pancreatic subcutaneous mouse tumors in vivo	NP delivers O_2_ to tumors and reduces tomor hypoxia Production of ROS to destroy tumors	[42]
Theranostic polymer microcapsules composed of hydrogen-bonded multilayers of tannic acid and poly(N-vinylpyrrolidone) loaded with DOX	5 µm	Th Dia	1/Diagnostic US: f = 2 MHz, i = 115 mW/cm^2^; 2/ Therapeutic US: f = 20 kHz, i = 15–257 W/cm^2^	50% DOX release from NM exposed to US → 97% destruction of MCF-7 human cancer cells in vitro (no cytotoxicity without US application) Capsules can be imaged under B-mode US	1/low intensity diagnostic US → 20–30% DOX release from NM 2/high intensity therapeutic US → up to 75% DOX release from NM	[65]
MB with Porphyrin Grafted Lipid (PGL) + Camptothecin-Floxuridine Conjugate (CF)	0,5–9 µm (MB) 30–100 nm (NB)	Th Dia	f = 1–7 MHz; i = 1 W/cm2; MI < 0.06	MB + ultrasound + laser leads to 90% tumor inhibition rate of HT-29 colorectal cancer with no recurrence in vivo	Transformation of MB into NB under US application produces accumulation of chemo-drugs/photosensitizer in tumors due to enhanced EPR effect PDT (laser) leads to reduction of (ATP)-binding cassette subfamily G member 2 (ABCG2) expression responsible for the drug resistance in chemotherapy	[162]
MB containing a core of PFP-Oxygen and a shell of PLGA loaded with ICG and PTX	150 nm (before laser irradiation) 700 nm (after laser irradiation)	Th Di	f = 1 MHz I = 1 W/cm^2^	MB exposed to laser + US: apoptosis of SKOV3 cells in vitro + SKOV3 tumor growth inhibition in vivo → MB can be monitored by US/PA and guided towards tumors	MB release PTX and generate ROS under laser and US exposure MB increase US contrast and improve PA imaging;	[163, 164]
MnOx/TiO-Graphene-polyvinylpyrrolidone Nanocomposite	260 nm	Th Di	f = 1 MHz; I = 1–1.5 W/cm^2^;	Complete eradication of 4T1 subcutaneous mouse tumors without re-occurrence following NM injection and US + laser administration; (60 °C during 600 s reached during treatment)	Association of TiO_2_ and graphene prevents electron/hole recombination upon US application and facilitates ROS generation; MnOx enables T1-weighted MRI	[64]
Hydrophilized Au-TiO_2_ nanocomposites	200 nm	Th	NA	Mice bearing SCC7 tumor injected iv with Au-TiO_2_ nanocomposites and exposed to US → ROS production + tumor suppression	Combination of TiO_2_ with Au improves ROS generation by TiO_2_	[51]
Au NP	15 nm	Th	HIFU i = 10–20 W; f = 1.2 MHz;	For US intensity of 10 W, the maximum temperature rise increased by 32% and 43% for Au NPs concentrations of 0.0625% and 0.125%, compared with the heating without NP; For US intensity of 20 W the lesion volume doubled and tripled for Au concentrations of 0.0625% and 0.125% compared with the heated volume without NP	Application of US in the presence of Au NP:larger temperature increase and larger heated volume than in the absence of NP	[46]
NB conjugated with folate (FOL)	287 nm	Th Di	f = 9 MHz; MI = 0.12;	MCF-7 over-expressing FR: enhanced targeting of NB and better US imaging;	Greater cellular targeting ability for (FOL)-NB than for non targeted NB	[25]
Naturally occuring heme-based pigment biliverdin NP	100 nm	Di	Photoacoustic imaging with Endra Nexus 128 photoacoustic tomographer (excitation between 680 and 800 nm)	NP injected in mouse leg: Detection of NP in lymph nodes using PA	NP strong absorbance at 365 and 680 nm. Excitation in near-infrared: PA signal Excitation in UV: fluorescence	[55]
PFP/C_9_F_17_-PAsp(DET)/CAD/PGA-g-mPEG ND	400 nm	Th Di	Di: 3.5 MHz, MI = 0.08;	Increased ND internalization in HepG2 and CT-26 cells following US application	ND: i) contrast agent in US imaging, ii) can release DOX at acidic pH, iii) efficient carrier due to cationic amphiphilic fluorinated polymer	[28, 29, 52]
Oxygen-deficient bimetallic oxide MnWO_X_ NP	6 nm	Th Di	f = 40 kHz; i = 3 W/cm^2^;	iv/it injection of NP in 4T1-tumor bearing mice → tumor growth retardation; NP metabolized without long-term toxicity	NP stable, biocompatible, produce more ROS than protoporphyrin IX and titanium dioxide, (MnWO_X_ traps electrons/prevents electron–hole recombination	[28, 29, 52]
Outer membrane vesicles (OMV) encapsulating biopolymer melanine	100 nm	Di	f = 5 MHz	It administration of OMV in subcutaneous 4T1 mouse tumors followed by laser irradiation (1.5 W/cm^2^, 800 nm, 6 min) → tumor disappearance	OMV produces heat under laser irradiation that can be used to destroy tumors and for PA imaging	[57]
PLGA-R837/PLGA-MPLA NP	100 nm	Th	HIFU (f = 4 MHz; i = 43 W)	Colorectal tumors (CT26) grown on both mouse flanks; tumor on one flank removed with HIFU therapy to remove the larger tumor; 40 days after treatment, NP injected and second tumor disappears	R-837/MPLA: agonists of TLR7/TRL4 HIFU → produces tumor antigen; NP → adjuvants stimulating immature DC and naive T cells at tumor sites and tumor-draining lymph nodes	[10]
Liquid perfluorocarbon (PFC) NP conjugated to 9E5 (antibody targeting epiregulin)	140 nm	Th	f = 5 MHz; Peak negative pressure = 1.5 MPa	NPs target 97.8% of EREG expressing cancer cells and kill 57% of those cells following US application; intracellular vaporization changes cell morphology;	Liquid PFC transformed in gas following US exposure; NPs conjugated to 9E5 selectively internalize in cancer cells; kill these cells by US-induced intracellular vaporization	[74]
DOX loaded human serum albumin NP attached at the surface of Chlorin e6 encapsulated MB	2500 nm	Th	f = 1 MHz; i = 0,2 W/cm^2^	NP/MB + US treatment reduces the number of cells with cancer stem-like cell property	Maximize anticancer efficiency by overcoming MDR NP/MB complex delivered to cells by sonoporation caused by MB cavitation ROS generated by intracellular delivered Ce6 under laser irradiation stops ABCG2 efflux receptor activity overexpressed in doxorubicin-resistant breast cancer cells (MCF-7/ADR), leading to improved chemotherapy efficacy	[36, 37, 38]
Membrane fusogenic liposomes loaded with Docetaxel (MFL-DCT)	100 nm	Th	f = 1.1 MHz; i = 20 W; focused	Mice bearing MDA-MB-231 tumors treated by iv injection of DTX-MFLs + MB followed by US application → tumor growth retardation	MFL-DCT can fuse with cell membrane and thereby efficiently deliver DCT inside cells;	[36–38]
pH-sensitive reduced albumin NP loaded with DOX	146 nm	Th	f = 1.1 MHz; i = 20 W; focused	Mice bearing MDA-MB 231 breast tumors injected with NP and treated by US: Tumor growth retaradation	Application of US improves the efficacy of EPR effect and the diffusion of NP in the tumor	[36–38]
Gaz vesicles (GV): gas-filled protein-shelled NC (produced intracellularly by certain bacteria/archaea	140–800 nm	Di	Imaging at 6 MHz (peak positive pressure of 0.3–1.2 MPa)	In vivo ultrasound imaging during passage through the inferior vena cava (IVC) in mice	US imaging of gas vesicles that can be genetically engineered	[58, 59]
Polymeric (poly(D,L-lactide-co-glycolide) NP containing calcium carbonate, where NP are bound to rabies virus glycoprotein (RVG) peptide (a targeting moiety to neuroblastoma)	220 nm	Di Th	f = 15 MHz (imaging);	Iv injection of NP in mice bearing N2a (neuroblastoma) tumors; NP accumulate at tumor site, NP increase US signals, NP reduce tumor growth	NPs produce carbon dioxide bubbles under acidic conditions (pH change) and enhance US signals NPs generating gas induces necrosis and reduces tumor growth	[169]
H_2_O_2_ encapsulated in Fe_3_O_4_-PLGA polymersomes	412 nm	Th Di	f = 40 MHz	Mice bearing HeLa tumors injected iv with polymersomes followed by US exposure display tumor disappearance	O_2_ is used as echogenic source for US imaing; OH production through the Fenton reaction by reaction of H_2_O_2_ and Fe_3_O_4_	[60]
Long-circulating lipid-coated MB	1500 nm	Di	f = 2.8 MHz	Mice bearing liver tumors injected with MB and SonoVue and imaged with US, MB stay in tumors longer than SonoVue and hence enable US contrast imaging for longer time	MB fabricated by changing the core of SonoVue microbubbles to a higher–molecular weight gas (C3F8) MB have smaller diameter and higher inertial cavitation threshold than Sonovue: better/longer organ imaging	[206, 207]
Liposomes co-encapsulating doxorubicin (DOX), hollow gold nanospheres (HAuNS), and perfluorocarbon (PFC)	200 nm	Th Di	f = 1.9 MHz	Mice bearing 4T1tumors injected iv with liposomes and tumors heated by laser irradiation (2 W/cm^2^); US signal detected in tumors; treatment leads to DOX release and increase of tumor temperature to 70 °C; tumor growth retardation	Heating of HAuNS by 808 nm NIR laser irradiation; Liposome tumor accumulation due to their nm sizes; Heating triggers DOX release; Gasification of PFC enhances US imaging signal;	[39]
Pt-CuS Janus NM composed of Pt and CuS with inner cavities loaded with sonosensitizers (tetra-(4-aminophenyl) porphyrin, TAPP)	285 nm	Th Di	f = 1 MHz i = 1 W/cm^2^	Mice bearing CT26 xenograft tumors treated by NM iv administration followed by 808 nm laser irradiation and US Almost complete tumor resection without obvious reoccurrence (heat at 70 °C); Photoacoustic (PA) imaging and NIR thermal imaging	Pt enhances photothermal performance Pt leads to nanozyme activity for transforming H_2_O_2_ to O_2_ and overcome tumor hypoxia NM leads to ROS production	[72]
MON@C: Mesoporous organo-silica nanoparticles (MON) containing catalases (c) inserted inside pores	150 nm	Th	f = 1.1–3.5 MHz; i = 70–100 W;	Nude mice bearing MB231 xenograft tumors injected iv with MON@C followed by HIFU: O_2_ production yields an increase in tumor ablated volume by 10 compared with HIFU treatment with MON (no O_2_ production)	MON@C transforms H_2_O_2_ into oxygen bubbles more efficiently than free catalase Bubbles amplify echo signal to guide HIFU surgery; Bubbles intensify cavitation effect under HIFU irradiations Cancerous region displays larger H_2_O_2_ concentration compared to the normal tissue (effect specific to tumor region)	[40, 130]
AML: Liposome containing: i) Anethole Dithiolethione (ADT) and hydrogen sulfide (H_2_S) pro-drug, embedded in lipid bilayer, ii) SPION in liposome core	166 nm (liposome) 7 nm (MNP)	Th Di	f = 18 MHz; B mode imaging	NB specifically target tumor in vivo following magnetic field application Intra-tumoral conversion of nanostructure to microstructure: better anticancer efficacy Transformation monitored by MRI/US imaging	AML generate intra-tumoral H_2_S bubbles used for tumor imaging and tumor destruction (gas emission) under HIFU AML exposed to magnetic field: significant inhibition of tumor growth	[40, 43, 130]
NC: GPC3 antibody linked to PEGylated nanometric-reduced-graphene-oxide associated with NB	700 nm	Th	F = 1 MHz; I = 1–3.5 W/cm^2^	NC inhibit HepG_2_ cells following US and laser applications	US application leads to NB destruction and an increase in NC concentration around the HepG_2_ cells Heat at 60 °C by laser irradiation helps to destroy tumor cells	[208]
NB associated with fluorescent dyes	112 nm	Di	F = 4,4 MHz; MI = 0,1	NB: efficient imaging of mouse breast tumor (higher concentration in tumor compared with commercially available US CA agent)	Dyes yield BRET − FRET mechanism and large increase in fluorescent signal NB enables imaging by US of delineation of tissue microvasculature	[26]
EXO-DVDMS: sinoporphyrin sodium (DVDMS), attached to tumor cell-derived exosomes (EXO)	150 nm	Di Th	f = 1 MHz; i = 3 W;	EXO-DVDMS followed by ultrasound applications: growth inhibition of breast cancer-lung metastasis	Exosomes serve as camouflage to enable DVDMS to reach tumor sites; Exosomes target specifically homotypic tumors US is applied to enhance accumulation of EXO-DVDMS is tumors (improved efficacy of EPR effect) EXO-DVDMS endocytosed by lysosomes; low lysosome pH yields DVDMS release and triggers cell death-signaling pathways	[56]
NP: (mPEG-PLGA) associated with anti-carcinoembryonic antigen and anti-carbohydrate antigen 19–9 encapsulating PTX	100 nm	Di Th	f = 1 MHz i = 1 W/cm^2^ UTMD (ultrasound targeted microbubble destruction)	NP have prolonged imaging time in rabbit kidney and tumor of nude mice compared with Sonovue NP have potential enhanced antitumor effect due to longer tumor residence time	NP internalization facilitated by the application of US	[209]
NC: PLGA NC coated with a thin Silica layer encapsulating i) perfluorocarbon (PFOB), ii) antitumor Ruhenium complex, iii) superparamagnetic Fe_3_O_4_ NP	225 nm	Di Th	i = 270 W (HIFU)	HeLa tumor-bearing nude mice injected iv with NC followed by HIFU treatment resulted in more tumor inhibition than HIFU alone	Imaging by US and MRI Image-guided HIFU-triggered chemotherapy PFOB/gas in NC causes collapse of the shell under HIFU and releases Ruhenium complex	[44]
Self-assembled peptide-based NP encasulating phalloidin	300/1200 nm	Di	NA	Internalization in A549 cells of phalloidin into cells following US-triggered rupture of nano-peptisomes	Phalloidin delivered to cell cytoplasm upon US-mediated rupture of nano-peptisomes	[63]
NC: Anti-cancer drug + PFP encapsulated in glycol chitosan NP Hydrophobic core/hydrophilic shell	432 nm	Di Th	i = 0.0676 W/cm^2^; f = 10 MHz; MI = 0.235;	Mice bearing SCC7 tumors injected IV with NC followed by US application: accumulation in tumor + tumor growth retardation (effect more pronounced than in the absence of US application)	NC echogenic due to MB formation US-triggered drug release Enhanced EPR effect (nanometric size)	[174]
Carbonate NP (pluronic external surface) + inner part: gaz/DTX (chemotherapeutic drug)	100–300 nm	Th Di	f = 10 MHz; MI: 0.235; i: 0.0676 W/cm^2^	Mice bearing SCC7 tumors injected iv with NC followed by US applications: tumor growth retardation	Generates bubbles under US application; Release drugs under US application;	[137]
MB loaded with porphyrin	NA	NA	F = 1 MHz	Extravasation of MB outside of bllod vessels in vivo under US exposure	Transformation of MB to NB (gaz loss + shell shedding)	[68–70]
TNB: NB linked to NPY (Y1 receptor ligand) + DOX	300 nm	Th	f = 1,5–10 MHz; MI = 0,7;	Mice bearing 4T1 tumors treated by iv injection of TNB + DOX followed by US application: tumor growth retardation;	DOX and TNB are injected separately; US irradiation favors EPR effect of DOX and DOX diffusion in tumor (with better efficacy than MB);	[210]
NC: hollow mesoporous TiO_2_ surrounded by DsDNA containing DOX	126 nm	Th	i = 1 W/cm2	Mice bearing MCF-7/ADR tumors injected iv with NC followed by US application: tumor growth retardation	Treatment enables to overcome drug resistance towards MCF-7/ADR via the inhibition of mitochondrial energy supply due to the “explosion” of NC Treatment produces ROS and releases DOX NC can escape lysosome following US application;	[50]
ND of PFC stabilized by albumin	160 nm	Th	f = 1 MHz; i = 3.5 W	Mice bearing 4T1 tumors injected iv with ND + liposomal Ce6 followed by laser and US irradiation: Tumor growth delay	NDs adsorb oxygen in lung + release oxygen in tumor under ultrasound stimulation (cycles are repeated); Enhances tumor oxygenation and improves efficacy of PDT/RT treatment of tumors	[30]
ND containing PFC, a photoabsorber/photoacoustic agent (PDI), and a photosensitizers ZnF_16_Pc	113 nm	Di	f = 40 MHz	ND injected iv in mice bearing U87MG glioblastoma: ND tumor accumulation via EPR effect (imaging of ND via PA/US following laser irradiation at 671 nm) PDT + PTT treatment, result in complete tumor eradication with minimal side effects	Laser irradiation of PDI: liquid-to-gas phase transition of PFC + PTT effect PFC provides O_2_ to tumor, hence improving PDT efficacy	[31]
Anti-EGFR-PEG-SPIO	9 nm	Th	f = 1–1.2 MHz i = 60–160 W/cm^2^	Treatment of rats with lung tumors injected with SPIO and exposed to US: tumor growth retardation	SPIO specifically target lung cancer cells overexpressing EGFR SPIO yield more heat in tumors than healthy tissues SPIO improve MRI sensitivity for visualization of EGFR overexpressed lung cancer in a rat model	[48, 49]
NET-1 siRNA-conjugated sub micron bubble (SMB)	600 nm	Th	f = 1 MHz; i = 1 W/cm^2^	NET-1 siRNA-SMB brought into presence of SMMC-7721 human hepatocellular carcinoma cells followed by low-frequency US application → enhancement of gene transfection efficacy	SMB exposed to low-frequency US promotes gene transfection	[211]
Echogenic NB enclosing cell-permeable peptides (CPPs) and siRNA	200 nm	Th	f = 1 MHz; i = 1 W/cm^2^	NB brought into presence of human breast tumor cells followed by US application: releases of 90% of encapsulated CPP-siRNA (compared with 1.5% without US) NB administered iv to mice bearing fibrosarcoma HT-1080 tumors: i) NB tumor accumulation, ii) increase in c-myc silencing, iii) tumor growth delay	Local ultrasound stimulation triggered the release of CPP-siRNA from the NBs and activated its tumor cell penetration	[170]
Peptide labeled semimetal bismuth NP (Bi-LyP-1 NP)	3,6 nm	Th Di	PA using a Vevo LAZR PA system	Mice bearing 4T1 tumors injected iv with Bi-LyP-1 NP exposed to laser + X-ray: i) heat at 45 °C during 15–20 min, ii) tumor growth retardation; Bi-LyP-1 NP: cleared from mice through renal/ fecal routes after 30 days	Bi-LyP-1 NP: more tumor accumulation with peptide LyP-1 than without LyP-1; Bi-LyP-1 NP: Absorb both ionizing radiation and the second near-infrared (NIR-II) window laser radiation; Bi-LyP-1 NP: dual mode imaging (CT/PA); Bi-LyP-1 NP: efficient synergistic NIR-II photothermal/radiotherapy of tumors	[53]
Silicon needle covered by Zinc-Oxide nanowires (ZnONW)	MB: 5–20 µm	Th	f = 20 kHz; i = 5 W/cm^2^;	Mice bearing MC4L2 tumors treated by iv injection of ZnONW followed by US application: ≈82% decrease in tumor size within 10 days compared with 25% with PTX	ZnONW produce MB under US application	[212]
NC: ammonium bicarbonate, gold nanorods and DOX encapsulated in folic acid conjugated liposomes	100–150 nm	Th	f = 1 Hz; i = 1 W;	S180 tumor-bearing mice injected iv with NC followed by laser or US application: tumor growth delay	NC allows: i) multimodal imaging (CT/US), ii) local release of drug (doxorubicin), iii) hyperthermia Ammonium bicarbonate allows controlled DOX release	[6]
NC: terrylenediimide (TDI) poly(acrylic acid) (TPA) based nanomedicine (TNM)	13 nm	Th Di	NA	4T1 bearing mice administered iv with NC followed by laser irradiation: tumor growth delay	Temperature increase under laser irradiation (up to 60 °C) PA imaging of NC in tumor	[213]
Polymersome embedding perfluorocarbon and DOX;	178–437 nm	Th Di	f = 1 MHz Acoustic pressure: 2 Mpa	Mice bearing C6 glioma tumors injected iv with NC and exposed to US: tumor growth retardation	Size of NC increases from 178 nm during circulation to 437 nm in acidic tumor microenvironment NC small size allows efficient tumor uptake NC swells at tumor size to become efficient CA for US imaging; NC release DOX in tumor under US application	[61, 62]
Biomimetic nano-system (BM): Macrophage membrane coated on CAu-BMSN: CORM-401 (H_2_O_2_-sensitive CO release prodrug) loaded into Au NP associated with black phosphorus quantum dots	50 nm	Th Di	f = 1 MHz; i = 1 W/cm^2^;	Mice bearing 4T1 tumors injected iv with biomimetic nano-systems followed by US application: tumor growth retardation	BN: tumor-targeted delivery of singlet oxygen (1O_2_) and carbon monoxide (CO) following US application in presence of H_2_O_2_ in tumor microenvironment; Tumor targeting favored by macrophage membrane (RES evasion) Cell apoptosis caused by mitochondrial dysfunction Effective immune responses through indoleamin 2,3-dioxygenase (IDO) signal blocking (prevents tumor regrowth and metastasis)	[75, 135]
Annexin V-conjugated NB (A-NB)	635 nm	Th Di	f = 10–60 MHz	Long lasting US imaging of NC in MDA-MB-231 mouse tumors	A-NB: extravasate in tumor vasculature and recognize apoptotic tumor cells A-NB: better tumor US contrast than non targeted NB	[214]
NC: MnOx with biocompatible/biodegradable hollow mesoporous organosilica NP conjugated with protoporphyrin (sonosensitizer) and cyclic arginine-glycine-aspartic pentapeptide (targeting peptide)	100 nm	Th Di	f = 1 MHz i = 1.5 W/cm^2^	Mice bearing U87 tumor xenograft injected iv with NC followed by US application: suppressed tumor growth NC can be imaged by MRI in tumor, enabling therapeutic guidance/monitoring during SDT	MnOx: i) nanoenzyme converting H_2_O_2_ (overexpressed in tumor) into oxygen, ii) increasing tumor oxygen level, iii) facilitating ROS production, iv) improving SDT efficacy	[71]

CA, contrast agents; CAV, Commercially available; CM, confocal microscopy; CT, computed tomography; DOX, Doxorubicin; ICG, Indocyanine green; MNP, magnetic nanoparticle; MB, Microbubbles; MI, Micelles

MNC, Magnetic nanocapsule; MPLA, monophosphoryl lipid A; NM, Nanomaterial; NB, Nanobubbles; NC, Nanocapsule; ND, Nanodroplet; NM, Nanomaterials; OMV, Outer membrane vesicle; PA, Photoacoustic; PFP, Perfuoropentane; PLGA, poly(lactic-co-glycolic acid); PTT, Photothermal therapy; PTX, paclitaxel; SPION, Superparamagnetic iron oxide nanoparticles; SS, Sonosensitizer; TPA, Two photon absorption; US, Ultrasound; XIAP, X-linked inhibitor of apoptosis protein

### Diffusion of nano-systems through physiological barriers favored by ultrasound application

To reach a tumor, nano-systems should cross certain physiological barriers, such as those that limit penetration through tissue or cell uptake, or induce variable circulation/perfusion, extravasation, [78]. These barriers protect the organism against invasion by external agents. The application of ultrasounds has first been shown to favor the diffusion of nano-systems through the skin, in particular the stratum corneum, [9]. This mechanism is particularly efficient using low frequency ultrasounds of typically 20 kHz that penetrate deeply through the skin. Its efficacy was reported to rely on a combination of cavitation [79, 80], thermal [81, 82], radiation force, convection [83], and lipid extraction [84] effects resulting from ultrasound application. Thus, nano-systems consisting of liposomes comprising siRNAs have been delivered to skin tumors through mouse epidermal and dermal layers under the application of 20 kHz ultrasounds. The treatment led to a reduction of melanoma tumors grown under the skin of the treated mice, [85]. The blood brain barrier (BBB) can also be temporarily disrupted by low frequency focused ultrasounds, resulting in efficient nano-system diffusion in the brain with a high precision, *i.e.* with a resolution below 1 mm, a phenomenon attributed to physical disturbances created by MB and to temperature increases, both effects yielding BBB permeabilization, potentially synergically, [86]. Hence, liposomes encapsulating DOX were shown to efficiently cross the BBB, leading to DOX delivery in the brain, [87].

### Mechanisms involved in anti-tumor activity

**Fig. 2:**
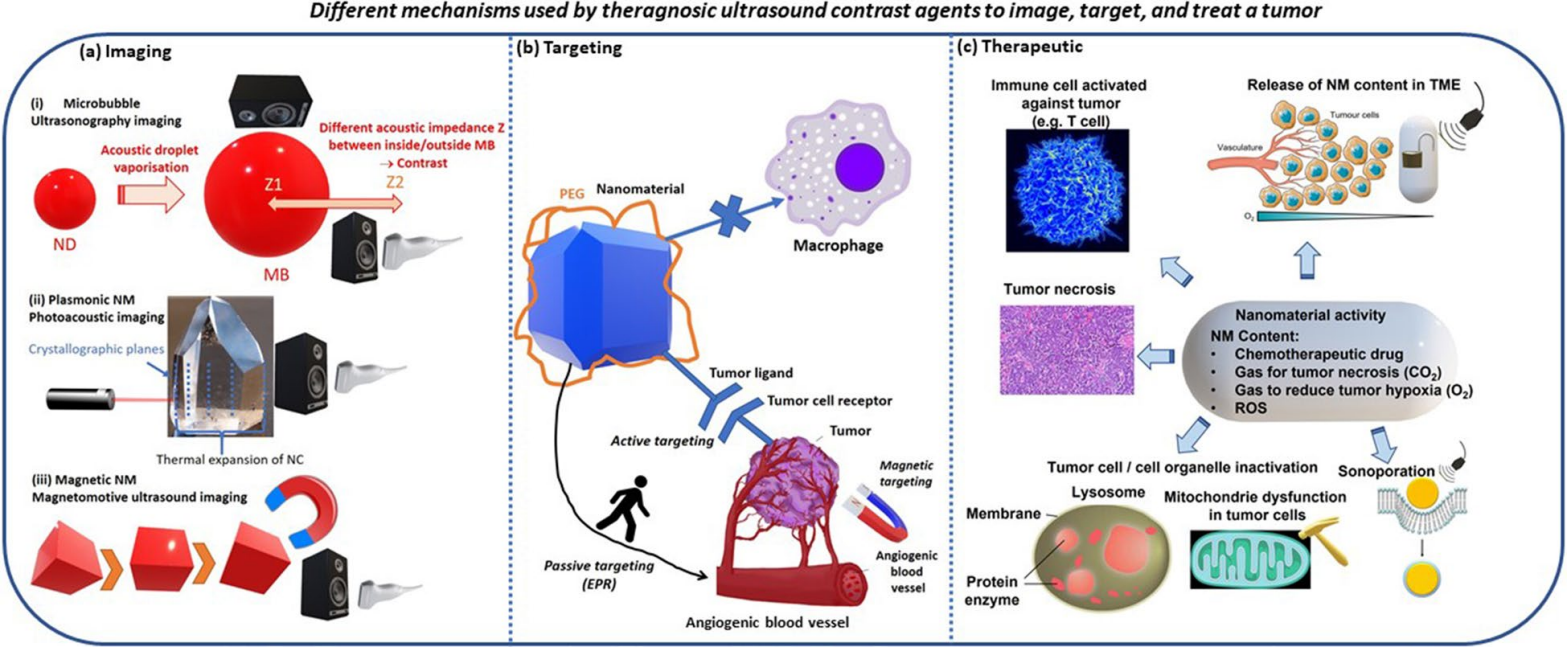
A summary of the various mechanisms following which nano-systems can become active for tumor targeting, imaging, and treatment

The various mechanisms of action responsible for the anti-tumor activity triggered by nano-systems exposed to ultrasounds are summarized in Fig. 2.

#### Heating

Heating can be beneficial in tumor treatment, e.g. through tumor ablation, increased blood vessel/tissue permeability, triggered drug release, [88]. Nano-systems (NS) could favor an ultrasound anti-tumor heat treatment in two ways. First, metal-based NS can increase the amount of heat produced by ultrasounds, [89]. Such mechanism may be attributed to the presence of nano-systems in the tumor, which enhances ultrasound attenuation at high ultrasound intensity and frequency, [90]. Thus, when Au, NGO or IONP NP were injected to tumors or Au NP were mixed in water and these mixed systems were exposed to ultrasounds, it yielded a more pronounced temperature increase by 15 to 40% for NP contained in tissue than for NP-free tissue, [45], and to a higher heating rate of 1.6 °C/min for Au NP dispersed in water than for free water, i.e. 0.4 °C/min, [45]. Such results were obtained for specific values of NP concentrations, i.e. 0.2 µg of NP per mm^3^ of tissue, ultrasound intensity, i.e. i = 1–2 W/cm^2^, and ultrasound frequency, i.e. f = 1 MHz, [90]. To the author knowledge, the range of values of the various parameters, i.e. NP size, composition, concentration as well as ultrasound intensity, and frequency, which should yield an optimal enhancement of the thermal efficacy in the presence of NS, have not yet been reported.

Second, NS can consist of temperature sensitive phospholipid or polymer membranes able to switch from a stable configuration at physiological temperature, where they maintain drug encapsulation, to an unstable state at a higher temperature resulting from ultrasound application, which leads to membrane destruction and drug release. The transition occurs at the so-called transition temperature, which is typically within the range of mild hyperthermia (40–43 °C). Thermosensitive liposomes can be composed of phosphatidylcholines, [91], PEGylated phospholipids associated to DPPC/DSPC, [92], porous lisolipids membranes, [93]. They can trigger anti-tumor activity through a combination of temperature triggered anti-cancer drug release /activation and mild hyperthermia, [91]. A drawback that has slowed down the development of these liposome lies in their relative instability, which can lead to their destruction before they reach the tumor, [94], and methods to overcome this lack of stability have been developed using DPPGn lipids that are not leaky at normal body temperature, [95].

#### Cavitation

The most frequently described mechanical effect that can generate anti-tumor activity under ultrasound application is cavitation. For the sake of clarity, it is common to separate it from the thermal effect described above. However, cavitation and thermal mechanisms are related to each other, i.e. cavitation can create a temperature increase while a temperature variation can affect cavitation. NS may act as nuclei of cavitation bubbles and therefore potentially enhance the level of cavitation resulting from ultrasound application, [9]. In general, two types of cavitation can be distinguished, *i.e.* stable and inertial ones. In stable cavitation, gas pockets are formed, which oscillate periodically in size through so-called acoustic compression and decompression cycles, which generate fluid streaming and mechanical stresses, which have been reported to be able to destroy cancer cells, [9]. By contrast, inertial cavitation is an unstable phenomenon, in which bubbles generated by ultrasounds expand and collapse, potentially leading to high temperatures (> 5000 K), pressures (> 800 atm), and ROS production, [9]. Nano-systems associated with cavitation phenomena have been reported to be either nano-bubbles or micro-bubbles, with some systems transiting between these two states. In general, two types of micro/nano bubbles can be distinguished, those endogenous whose formation results from ultrasound application, and those exogenous that are administered on purpose in the organism. The existence and nature of cavitation therefore does not only depend on the type of nano-system, but also on ultrasound parameters such as ultrasound frequency, pressure, intensity, pulse sequence, or duration of application. Thus, the behavior of micro/nano bubbles was reported to depend on negative acoustic pressure, which can trigger nano/micro bubbles growth or expansion. While a relatively low negative acoustic pressure associated with mechanical indexes MI of typically 0.1 < MI < 0.3 could favor stable cavitation, a higher negative acoustic pressure associated with MI larger than typically 0.3–0.6 may more easily result in nano/micro bubble disruption and unstable cavitation, [96]. In fact, the formula, which provides an expression of MI as the ratio between the negative acoustic pressure and the square root of the ultrasound frequency, [5], does not seem to take into account other parameters that can influence MI values, such as: (i) the medium through which ultrasounds travel, i.e. for the same acoustic parameters, the mechanical index was shown to differ in water and blood, [97], or (ii) the acoustic wave pulsation length, [98]. Furthermore, it was also reported to use a high ultrasound frequency to reduce MI and avoid unstable cavitation, [98]. As an example, polymeric NP containing porphyrins, which were exposed to ultrasounds of frequencies maintained below 20 MHz and high-pressure of amplitude up to 120 MPa were shown to induce cavitation on an in-vitro neuroblastoma model, [99]. Ultrasound MI is often varied to obtain the desired treatment mechanism. The effects of cavitation are thus diverse, ranging from the destruction of cells to the creation of pores in or between cells, to the enhancement of endocytosis, depending on the ultrasound setting parameters, [100]. One of the main challenges with cavitation lies in its highlighting through the visualization or measurement of the bubbles that it generates, specifically in an organism, which is a difficult task, [101]. Some studies have overcome this difficulty, publishing results on cavitation monitoring, [102]. Furthermore, clinical trials were launched, which used cavitation as an underlying mechanism, [103].

#### Sonoluminescence

Sonoluminescence (SL), which results from cavitation, is characterized by flashes of light emitted by cavitation bubbles, [104]. It can possibly result in the apparition of an electron/hole pair and subsequently the generation of ROS species. For example, when microbubbles were produced by the application of ultrasounds, nonthermal cavitation was reported to occur, possibly resulting in sonoluminescent light exciting C-doped TiO_2_ NP. When these NP decayed back to their ground state, energy could be transferred to oxygen to generate ROS, which might then trigger tumor cell death, [105]. However, such cascade mechanisms should remain hypothetical in the absence of a method enabling to directly measure or observe them. Sonoluminescence may also be responsible for the activation of light-sensitive drugs (LSD) through the light that it triggers. Hence, it creates a bridge between Photodynamic and Sonodynamic therapy, where Sonodynamic therapy displays the advantage of exciting LSD at a larger depth than PDT, since ultrasounds usually penetrate more deeply than laser light, [104].

#### Sonoporation

Although separated from the cavitation section for the sake of clarity, sonoporation, which may also be designated as sonopermeation, could be associated with the formation of pores in cell membranes under ultrasound exposure and therefore result from cavitation, [106]. It can favor the diffusion of NS through the cell membrane, via a mechanism called Sonoporation, [107–109], enabling the capture of NS and their associated functional elements by tumor cells. This mechanism can be triggered by: (i) intracellular interactions between microbubbles trapped inside cells and cell membranes, (ii) micro-jetting, (iii) micro-streaming, (iv) shock-waves, and (iv) diffusion of microbubbles through cells, [9]. Intracellular interactions can be characterized by backward and forward movements of microbubbles, which apply a mechanical force on cell membrane that is sufficiently strong to permeabilize it. In general, jets, streams, shockwaves occur under ultrasound application even in the absence of micro-bubbles, [110]. The addition of micro-bubbles should result in the location of these mechanisms around or near these bubbles. Micro-streaming is associated with the fluid movement around bubbles, resulting from the energy being transferred from the ultrasounds to the fluid. In some cases, sonoporation was reported to be a relatively mild effect, due to non-inertial cavitation, which enhances cell permeability and favors NS or drug displacement without significantly damaging cells, [111], enabling for example blood vessel permeabilization, [112–114]. In some other cases, sonoporation was described as a mechanism inducing cytotoxicity and the mechanical destruction of cell membrane in the presence of NP, [115, 116], possibly due to NP interactions with cell membrane, [117, 118]. Furthermore, sonoporation mechanism was reported to occur for a wide range of different ultrasound frequencies, *i.e.* typically comprised between 0.02 and 6 MHz, [119, 120], and low intensities, *i.e.* typically below 1 W/cm^2^, [120], suggesting that this method can relatively easily be transposed clinically, [121], given the easily achievable ultrasound parameters that it requires.

#### Controlled drug release from nano-systems under ultrasound application

When they are used without being exposed to a source of radiation, NS often suffer from a lack of control over the anti-tumor activity that they trigger. Such drawback can be overcome by exposing certain NS to ultrasounds to achieve drug release/activation on demand. For example, it was shown that drugs/bio-active molecules could be released from polymer-based NS, [122], hydrogels, [123], microbubbles following ultrasonic excitation, leading to significant drug accumulation in tumor tissues and then to tumor growth inhibition, [124]. Such mechanism displays a number of advantages. It allows repetitive drug release by applying ultrasounds several times, [123]. It prevents permanent drug damages by using mild ultrasound treatment parameters. It prevents drug release in the absence of ultrasound application. It leads to a reduction of detrimental side effects of toxic chemotherapeutic drugs. It decreases drug leakage in blood circulation. It yields controlled drug release in the tumor, hence limiting healthy tissue exposure [125–128].

#### Gases activated against the tumor

Certain gases have been reported to be involved in anti-tumor activity, [129]. Firstly, they could be inserted inside nano/micro bubbles, and released in a controlled manner in the tumor under ultrasound application. Secondly, they could undergo a transformation to become active, e.g. through in situ conversion of H_2_O_2_ to O_2_. In fact, the tumor micro-environment often displays high levels of H_2_O_2_, which can be converted to O_2_ by catalase enzymes or inorganic systems mimicking enzyme activity, as observed for catalase associated to iron oxide nanoparticles that produced O_2_ bubbles in tumors under HIFU application, [130]. The way in which gases act against the tumor depends on their nature. When present in tumor, Oxygen (O_2_) can help overcoming tumor hypoxia, which undermines chemotherapy efficacy, nitric oxide (NO) can yield the production of highly reactive ROS, i.e. peroxynitrites (ONOO^−^), [131–133], hydrogen sulfide (H_2_S) can lead to selective ROS activation, [134], sulfur dioxide (SO_2_) can help regulating redox balance in tumor, [75, 135], CO_2_ can favor drug release, [136]. The use of NS to deliver gases enables overcoming the problem associated with their low solubility and irritability through their encapsulation in nano/micro bubbles. Moreover, when they are associated with nano-systems, gases can either be selectively released in the tumor microenvironment (TME), [137], or be produced in TME, [138]. Such gases can also help to visualize and release drugs, [139], to starve tumor cells through CO generation that blocks nutrients, *e.g.* glucose or O_2_, [140], or to create tumor embolization using nano-bubbles filled with SF_6_ and thrombin exposed to ultrasounds, [141].

### Micro to nano or nano to micro size conversion

One of the most appealing features of treatments combining ultrasound and NS comes from the adjustment of NS sizes that they allow. This is essentially achieved with nano/micro bubbles/droplets, which can either increase in size from being nanometric to being micrometric or inversely decrease in size from being micrometric to being nanometric, [142]. On the one hand, such mechanism can be due to the mechanical nature of the acoustic wave, which enables applying a positive/compressive and/or negative/expansive pressure on these NS. On the one hand, it can come from vaporization of nanodroplets, i.e. a transition from a liquid to a gaseous state of the droplet that yields its expansion, [143, 144]. While the first mechanism is purely acoustic, the second one can be generated by other radiations than ultrasounds such as laser, [145, 146]. Such duality in NS sizes enables bubbles/droplets to combine a good echogenicity achieved under micrometric sizes with a faculty to passively diffuse towards the tumor via EPR effect resulting from their nanometric dimensions, [147]. For example, a nanodroplet made of biodegradable block copolymer of PEG/PLLA with a low boiling temperature of 29 °C encapsulating DOX and PFP, displayed the capacity to: (i) vaporize upon heating to physiologic temperatures and be transformed into a microbubble acting as ultrasound contrast agent, (ii) extravasate into a mouse tumor following its iv injection due to its nanometric size during its transport towards the tumor, (iii) release DOX in the tumor to yield significant chemotherapeutic efficacy, [148]. At the opposite of this transformation, lies the transition of MB from micrometric to nanometric sizes. As a first example, MB consisting of porphyrin-phospholipid shell encapsulating perfluoropropane gas can transform into smaller porphyrin NP under ultrasound application, and lead to high accumulation of porphyrin at tumor site, [149]. As a second example, MB loaded with siRNA could be converted into NP upon ultrasound exposure, hence leading to efficient anti-tumor efficacy among tumor-bearing mice receiving this MB/ultrasound combined treatment, an effect which was attributed to an improved XIAP gene silencing and cleaved caspase-3 activation, [150]. In order for these methods to be fully efficient, ultrasounds should be applied at the right moment, i.e. after and not before bubbles/droplets have reached the tumor, and a too strong or stringent interaction between ultrasounds and bubbles/droplets should be avoided to prevent their destruction, [151].

### Preclinical studies preceding the use of nano-systems in the clinic

**Fig. 3:**
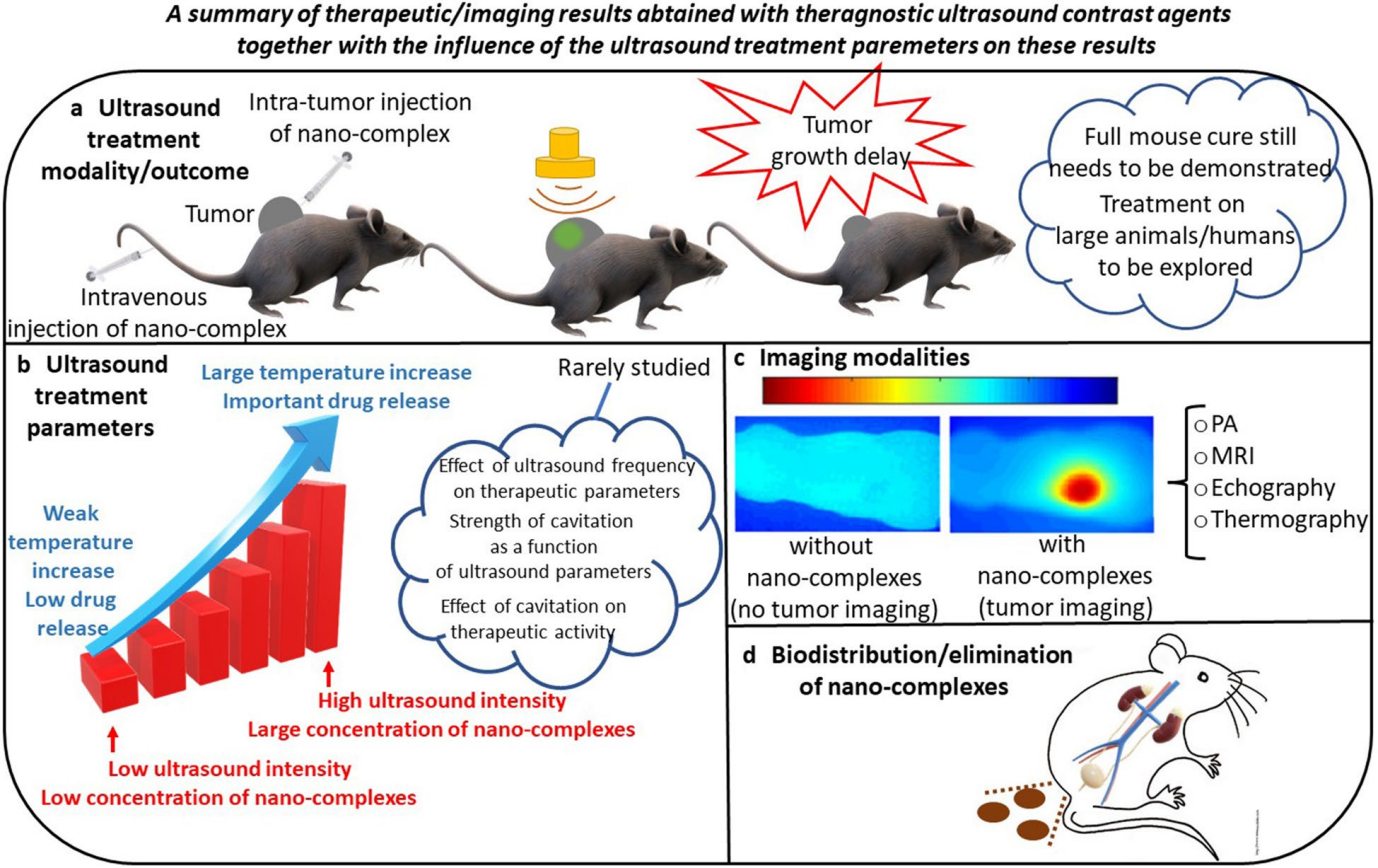
Schematic diagrams representing: **a** the therapeutic outcome of tumor treatments obtained on mice administered with nano-systems and exposed to ultrasounds, **b** the strength of various parameters (temperature increase, drug release, ROS production) influencing anti-tumor activity as a function of ultrasound intensity, as reported in some specific cases, **c** the different imaging modalities that could be implemented during a cancer treatment with NS exposed to ultrasounds, **d** NS biodistribution ending by NS elimination, as reported in some specific cases

The clinical use of nano-systems could be foreseen to improve the imaging and/or therapeutic ultrasound capability. Such prediction largely relies on preclinical data, which have been obtained. Figure 3 presents several schemes, which were drawn based on preclinical results. They illustrate the ways in which NS could be used to treat and image a tumor following ultrasound application, which parameters may influence ultrasound treatment efficacy, and NS biodistribution potentially yielding NS elimination following treatment.

#### In vivo tumor imaging

NS could be used for ultrasound tumor imaging. First, microbubbles, which are commonly used ultrasound contrast agents, [152], may enable super resolution ultrasound microscopy, [153]. For example, MB were sent to blood vessels irrigating tumors, they could serve as US contrast agents to image these vessels with a resolution, which is below the acoustic diffraction limit, [154]. Second, various nano-systems can be excited by laser light, producing their thermal expansion and an acoustic wave, which is further detected by an ultrasound detector, a method called photo-acoustic (PA) imaging that can potentially be applied for the visualization of deep tumor tissues, [155]. Advantageously, this method can easily be combined with laser-based tumor treatment techniques such as PDT and PTT. For example, tumor-bearing mice, which received intravenously DPP-TPA NP followed by passive tumor targeting of the NS as highlighted through PA imaging, displayed the successful destruction of their tumor via PDT and PTT treatments, [156]. Third, the use of NS enables combining echography with other imaging methods such as CT or MRI, [157]. Thus, mice bearing hepatocellular carcinoma (HCC) tumors injected intravenously with ND loaded with iodine could be followed by ultrasound and CT imaging, where the presence of iodine enabled reaching a CT resolution close to that of MRI, [27]. This result appears important since CT is often left besides in the profit of MRI due to its lack of resolution. However, given CT low cost, easiness to use, and frequent implantation in hospitals, there may be an interest to remedy this situation by using such NS.

Whereas MB remain the main types of NS described for in vivo tumor imaging, recent studies have described the emergence of new NS usable as US contrast agents such as silica-based micro/nanoplatforms, [158], gas-stabilizing nanoparticles, [159], re-chargeable nanobubbles on amine-functionalized ZnO nanocrystals, [54], polytetrafluoroethylene nanoparticles, [160], where cavitation induced by exposing such NS to ultrasounds can possibly lead to enhanced ultrasound contrast.

#### In vitro anti-tumor efficacy

Combined NS/US treatments were tested in vitro on different cell lines to determine the values of the parameters, i.e. NS concentration, NS incubation time, ultrasound intensity and frequency yielding optimal tumor cell destruction, pore formation and endocytosis, [161]. Thus, tumor cell death was reported to increase with increasing quantity of NS, for NS concentrations comprised between 0.004 and 0.4 µg/mL, [34], 0.05 and 8 µg/mL, [6], 0.06 and 12 µM, [162], or 12 and 200 µg/mL, [52], with longer incubation time, typically varied between 24 and 72 h, [144], with increasing ultrasound intensities, typically comprised between 1 and 6 Watt, [56]. An interesting in vitro study reported that a decrease of ultrasound frequency from 55 to 40 MHz resulted in an enhancement in the production of ^•^OH and a decrease in cell viability from 20% at 55 MHz to 13% at 40 MHz, suggesting that the adjustment of the ultrasound frequency does not only affect the ultrasound penetration depth, but also the production of ROS. However, such observations were made at rather high frequencies. It is not certain that such behaviors would occur at lower frequencies (< 10 MHz), which are more frequently used in therapy. Furthermore, several studies reported an enhanced cytotoxicity when cells were exposed to both laser and ultrasound instead of ultrasound alone, [68–70], suggesting the existence of a true synergy between these two types of radiations, which may be attributed to their different and complementary contributions. Ultrasounds may favor mechanical displacements of NS or the bubbles/gas that they generate while laser might induce plasmon resonance waves at the NP surface. However, these mechanisms appear not to be comparatively discussed in the literature. In vitro cytotoxicity experiments were also used to highlight certain mechanisms of tumor cell death induced by these combined NS /US systems, such as NS internalization, [28, 29, 52], specific tumor cell targeting, [32], chemotherapeutic drug release, [129], or cellular apoptosis, [163, 164]. A very interesting study attempted to correlate the level of cavitation induced by US application with the damage that it could produce at cellular level, [165]. Gels mimicking tissues were filled with NS and exposed to HIFU, yielding MB observed by phase array scanning probe measurements. Similar treatments applied to tissues led to tissue ablation with a sharp increase in the ablated volume and a decrease in the peak negative pressure necessary to induce tissue ablation in the presence of NS. Hence, an indirect cause-and-effect link between cavitation and tumor tissue ablation was established, [165]. Recently, a nano-scalpel effect was reported as resulting from acoustic shock waves and associated mechanical damages applied on adenocarcinoma or leukemia cancer cells in the presence of various metal oxide nanoparticles, [166–168].

### In vivo anti-tumor efficacy

Anti-tumor activity of different nano-systems has been assessed on various subcutaneous tumors, i.e. essentially glioblastoma U87, [24], ovarian SKOV3, [31], breast 4T1/MDA-MB-231, [34], head and neck SCC7, [51], cervical HeLa, [44], neuroblastoma N2a, [169], colon CT26, [35], fibrosarcoma HT-1080, [170], tumors of typical sizes of 15 to 300 mm^3^, which were injected with 0.1 to 2 mg of NS and exposed to ultrasounds of various parameters, 10 min to 24 h following NS injection, (Table 1). The values of the ultrasound frequency and intensity used during the treatments vary a lot depending on the study, i.e. 40 kHz < f < 12 MHz and 2 W/cm^2^ < i < 257 W/cm^2^ (Table 1). It seems that low frequency/intensity favor sonoporation, [171], or drug release, [172], whereas high frequency/intensity are more likely to be used when a temperature increase is desired, [173]. Furthermore, ultrasound frequency and intensity will affect the depth of ultrasound penetration, as well as other parameters such as the size, number, type of bubbles, or amplitude of temperature increase, where these other parameters also depend on each other and on the medium through which ultrasounds travel, the distance between the tumor and transducer surface, the geometry of the transducer determining ultrasound trajectory, as well as the nature or composition of nano-systems. The parameters associated with NS injection and ultrasound application are summarized in Table 1. While most studies employed an intravenous injection, it was shown in one case that anti-tumor efficacy could be reached at a lower NS dose (by a factor of 4) using an intratumor instead of an intravenous injection mode, [52], suggesting that intratumor injection can potentially yield anti-tumor activity at lower NS injection dose than intravenous injection. The most described short-term treatment outcome is growth tumor retardation observed within 15 min to 30 days following the beginning of the treatment. To the author knowledge, the absence of tumor regrowth following treatment was not reported. It may however be achievable under conditions of optimized treatment parameters. It was deduced from preclinical studies that anti-tumor activity was due to controlled drug release in the tumor, [174], ROS production, [51], release or generation of gases in the tumor, [60], an increase of tumor temperature, [53], anti-tumor immune reactions, [75], or a combination of several of these mechanisms, [39]. Although some studies report cavitation measurements on small animals, [175], the in vivo detection of cavitation can be difficult, notably due to small gas bubbles that cavitation may create in vivo. In some studies, ultrasounds were only used to image the tumor in the presence of NS, and the treatment was carried out by applying other types of irradiations such as the laser for PDT, [30, 31], or PTT, [31]. To highlight immune mechanisms, two approaches were followed. First, CT26 subcutaneous tumors were grown on two mouse flanks. NS containing immune modulator MPLA or imiquimod were used as adjuvant and injected in the primary tumor located on a first mouse flank. This tumor was then exposed to ultrasound and radiofrequency to yield thermal ablation, *i.e.* tumor temperature above ~ 60 °C. It induced an immune response characterized notably by the activation of dendritic and long-term immune memory T cells and de-activation of T_reg_ cells, which resulted in a size reduction of the untreated tumor located on the second flank 80 days following treatment of the primary tumor, [10]. Second, 4T1 subcutaneous metastatic tumors received iv a NS able to produce ^·^O2 and CO in the tumor, which leads to tumor cell apoptosis, as well as an immune agent NLG919 that blocks the indoleamin 2,3-dioxygenase signal pathway. Such combined treatment resulted in T cell activation and T_reg_ inhibition, which prevented the growth of lung metastases, [75].

### Clinical uses of nano/micro-systems exposed to ultrasound for tumor treatment

One of the most striking features of medical ultrasounds lies in the large variety of tumor types that they allow to treat using a broad range of different approaches. Ultrasound parameters are usually adjusted to reach the desired effect, e.g. to typical intensity/frequency values of i ~ a few mW/cm^2^ and f ~ 1.5–50 MHz for tumor imaging, [176], i < 1 W/cm^2^ and f ~ 0.5–1 MHz for permeabilization of BBB, [177], and i ~ 10^3^ to 10^4^ W/cm^2^ and f ~ 20 kHz to 200 MHz for HIFU tumor destruction, [178]. Other advantages of ultrasounds come from the moderate cost and compactness of most equipment used to generate them compared with CT, MRI, and radiation therapy apparatus, [179]. Thus, a large number of ultrasound clinical applications have been developed in the oncology field, which are at various stages of development. They include: (i) the improvement of current tumor detection methods such as mammography, [180, 181], to either replace or complement them, (ii) the imaging of tumors before proceeding to a cancer treatment such as surgery, [182], (iii) the visualization of recurrent lymph nodes following head and neck tumor treatment, [183], which paves the way towards the use of echography to monitor tumor recurrence, (iv) the guidance of surgery or cryoablation during breast cancer operation to ensure that the treatment takes place in the right location, [176, 184], (v) the disruption of blood–brain barrier to yield enhanced chemotherapeutic drug delivery to brain tumor, [177], (vi) the production of heat within tumors using HIFU, resulting in ablation of prostate, [178], breast, [185], or brain tumors, [186], (vii) the use of endoscopic ultrasound to guide thermal ablation during pancreatic cancer treatment, [187], and (viii) the exposure of microbubbles containing nano-medicines to enhance the delivery and release of such drugs in the tumor, [88].

To improve the imaging resolution of standard echographs, ultrasound contrast agents (UCA) have been developed. The commercialized ones consist of 1 to 4 µm in diameter vesicles, which are stabilized by an external layer made of proteins (Optison) or phospholipids (Definity or Sonovue), filled with a gas acting as a contrast agent, which is either C_3_F_8_ for Option and Definity or SF_6_ for Sonovue, [188]. Most interestingly, some studies have shown that such contrast agents could be used not only to improve ultrasound imaging resolution, but also the efficacy of anti-cancer drugs, as demonstrated when gemcitabine was injected in combination with US treatment, leading to enhanced anti-tumor activity compared with a treatment using gemcitabine alone, [189]. It was also shown in clinical trial NCT02343991 that the disruption of the BBB in the presence ultrasounds could be facilitated in the presence of MB, improving the delivery of DOX to brain tumors. Clinical trial NCT02181075 involving patients with liver tumors showed that administration to these patients of thermosensitive liposomes encapsulating DOX (ThermoDOX) followed by ultrasound application successfully enhanced the amount of DOX accumulating in the tumor, [190].

### Applicability of Ultrasounds Contrast Agents for treating various cancers and paths towards clinical trials

Combining ultrasounds with nanomaterials in a cancer treatment is an approach, which is at a more advanced stage than it appears. Indeed, different types of therapeutic ultrasounds are already used in the clinic such as HIFU employed to treat liver, [190], thyroid, [191], breast cancer, [185], or low-intensity ultrasound to permeabilize the BBB, enable the diffusion of anti-cancer drugs through this barrier, and then let anti-cancer drugs target brain tumors such as glioblastoma, [191]. In addition, various nanomaterials are already injected into humans and used clinically, i.e. mainly iron-based NP and liposomes, [192]. Finally, the combination of ultrasound contrast agents with therapeutic ultrasounds has been tested, for example by exposing MB to HIFU to treat pancreatic tumors, [189], or for enabling drug release from liposomes in tumors, [193]. Treatments combining nanomaterials and therapeutic ultrasounds therefore hold great promise. To make them succeed, one can rely on regulatory developments, which have already enabled clinical validation of therapeutic ultrasounds and/or NM and choose a suitable combination of NM and ultrasound modality that should achieve a favorable benefit risk ratio based on preclinical predictions. This combined approach should improve the benefit/risk ratio in various ways depending on the type of UCA/US pair that is chosen, i.e. notably by enabling moderate tumor heating, specific tumor targeting of the anti-tumor active principle, and localized treatment at TME location. Such approach should yield a more efficient and less toxic cancer treatment compared with the use of ultrasounds or NM alone.

## Conclusion

Here, the use of various combinations of ultrasounds and nano-systems for cancer treatment has been reviewed. Whereas ultrasounds of low intensities are privileged for imaging to avoid heating effects, i.e*.* typically a few mW/cm^2^, more intense ultrasound beams are selected for tumor treatment, i.e. typically of a few W/cm^2^ for treatment involving moderate heating and up to 1000–5000 W/cm^2^ for HIFU. While high frequencies are often used to improve imaging resolution or the level of ultrasound beam focalization, low frequencies are chosen to achieve high penetration depth of ultrasounds in tissues. Ultrasounds alone are already largely used in the clinic, e.g. to carry out biopsies of tumor tissues using apparatus such as ExactVu, [194], or for treatments of prostate, [178], or breast tumors, [195], with HIFU. These methods can be further improved by using nano-systems, which should enable: (i) a better imaging sensitivity, [196], (ii) an enhancement in the magnitude of the temperature increase, [197], (iii) a better anti-tumor efficacy through the various anti-tumor mechanisms that nano-systems can trigger such as cavitation or the delivery in the tumor of a chemotherapeutic drug, special gases, heat, or ROS, (Table 1). The diversity of these mechanisms is not only due to the mechanical nature of the ultrasound beam but also to the existence of various types of nano-systems, which behave differently, e.g. nano/micro bubbles yield ultrasound contrasting imaging properties while liposomes enable chemotherapeutic drugs to be either encapsulated within their core or attached at their surface. With nano-systems, it is also possible to achieve therapeutic activity locally at tumor site. This can be due to NS targeting tumors through passive, active, or magnetic targeting, or to NS crossing certain physiological barriers such as the BBB by permeabilizing them. It can also come from NS transiting from a nanometric size, enabling NS to passively target tumors, to a micrometric size, employed for high resolution ultrasound imaging. Finally, it may arise from anti-tumor compounds being released or activated from NS under ultrasound activation or from NS being incorporated in tumor cells by sonoporation. A large number of pre-clinical studies carried out on tumor bearing mice have validated these approaches, demonstrating a more pronounced anti-tumor activity using a combined ultrasound/nano-system treatment than ultrasound or NS alone. Clinical trials are ongoing, notably with Thermodox, to further validate the use of such combined therapies in humans, [198].

A series of NS could be used for ultrasound application, deriving from those that are already approved for human injection, i.e.: (i) Acuitas ALC-0315 liposomes used as adjuvants in COVID-19 vaccines, [199], (ii) iron oxide NP, e.g. Venofer, used for the treatment of iron anemia disease, [200], (iii) iron oxide NP, e.g. Nanotherm, employed for magnetic hypermetherma treatment of solid tumors, [201], or Ferumoxytol serving to enhance MRI contrast, [202], (iv) Au NP such as Auroshell for the treatment of tumors by PTT, [203], (v) Microbubble such as Definity for enhancing ultrasound contrast, [204]. The existence of several NP compositions/structures compatible with human injection bodes well for the development of new ultrasonic contrast agents.

## Data Availability

Full availability and data and material.

## Abbreviations

ADV: Acoustic droplet vaporization
AU: Gold
BBB: Blood brain barrier
CT: Computed tomography
DOX: Doxorubicin
DPPC: 1,2-Dipalmitoyl-sn-glycero-3-phosphocholine lipids
DPP: Diketopyrrolopyrrole polymer
DSPC: 1,2-Distearoyl-sn-glycero-3-phosphocholine lipids
EGFR: Epidermal growth factor receptor
EPR: Enhanced permeability and retention effect
f: Ultrasound frequency
HER-2: Human epidermal growth factor receptor 2
HIFU: High intensity focused ultrasound;
i: Ultrasound intensity
IONP: Iron oxide nanoparticles
iv: Intravenous
it: Intratumoral
LIU: Low intensity ultrasounds
MB: Microbubbles
MI: Mechanical index
MRI: Magnetic resonance imaging
MPLA: Monophosphoryl lipid A
ND: Nanodroplet
NGO: N-doped graphene oxide
NM: Nanomaterial
NP: Nanoparticles
NS: Nano-systems
PA: Photo-acoustic imaging
PDT: Photodynamic therapy
PTT: Photothermal therapy
PEG: Polyethylene glycol
PFC: Perfluorocarbon gas
PGL: Poly(lactic-co-glycolic acid) copolymer
PLGA: Poly(lactic-co-glycolic acid) copolymer
PLLA: Poly (L-lactide) polymer;
ROS: Radical oxygen species;
TAPP: Tetra(4-aminophenyl)porphyrin
TME: Tumor micro-environment
TPA: Two photon absorption
UCA: Ultrasound contrast agent
US: Ultrasounds
XIAP: X-linked inhibitor of apoptosis protein
